# Using Kinect to classify Parkinson’s disease stages related to severity of gait impairment

**DOI:** 10.1186/s12859-018-2488-4

**Published:** 2018-12-10

**Authors:** Lacramioara Dranca, Lopez de Abetxuko Ruiz de Mendarozketa, Alfredo Goñi, Arantza Illarramendi, Irene Navalpotro Gomez, Manuel Delgado Alvarado, María Cruz Rodríguez-Oroz

**Affiliations:** 1 Centro Universitario de la Defensa, ctra. Huesca, Zaragoza, Spain; 2 University of the Basque Country (UPV/EHU), Paseo Manuel Lardizabal 1, Donostia-San Sebastian, 20018 Spain; 3 Neurodegenerative Disorders Area, Biodonostia Health Research Institute, Begiristain Doktorea Pasealekua, Donostia-San Sebastian, 20014 Spain; 4 CIBERNED, Network Center for Biomedical Research in Neurodegenerative Diseases, Madrid, Spain; 5 Donostia University Hospital, Donostia-San Sebastian, Spain; 6 Ikerbasque, Basque Foundation for Science, Donostia-San Sebastian, Spain; 7 BCBL, Basque Center on Cognition, Brain and Language, Donostia-San Sebastian, Spain; 8 Neurology Department, University Hospital Sierrallana. Neuroimaging Unit, Valdecilla Biomedical Research Institute, IDIVAL, Santander, Spain; 9 Department of Neurology University of Navarra Clinic, Pamplona, Spain

**Keywords:** Kinect, Classification methods, Bayesian networks, Parkinson disease, Gait

## Abstract

**Background:**

Parkinson’s Disease (PD) is a chronic neurodegenerative disease associated with motor problems such as gait impairment. Different systems based on 3D cameras, accelerometers or gyroscopes have been used in related works in order to study gait disturbances in PD. Kinect ^*Ⓡ*^ has also been used to build these kinds of systems, but contradictory results have been reported: some works conclude that Kinect does not provide an accurate method of measuring gait kinematics variables, but others, on the contrary, report good accuracy results.

**Methods:**

In this work, we have built a Kinect-based system that can distinguish between different PD stages, and have performed a clinical study with 30 patients suffering from PD belonging to three groups: early PD patients without axial impairment, more evolved PD patients with higher gait impairment but without Freezing of Gait (FoG), and patients with advanced PD and FoG. Those patients were recorded by two Kinect devices when they were walking in a hospital corridor. The datasets obtained from the Kinect were preprocessed, 115 features identified, some methods were applied to select the relevant features (correlation based feature selection, information gain, and consistency subset evaluation), and different classification methods (decision trees, Bayesian networks, neural networks and K-nearest neighbours classifiers) were evaluated with the goal of finding the most accurate method for PD stage classification.

**Results:**

The classifier that provided the best results is a particular case of a Bayesian Network classifier (similar to a Naïve Bayesian classifier) built from a set of 7 relevant features selected by the correlation-based on feature selection method. The accuracy obtained for that classifier using 10-fold cross validation is 93.40%. The relevant features are related to left shin angles, left humerus angles, frontal and lateral bents, left forearm angles and the number of steps during spin.

**Conclusions:**

In this paper, it is shown that using Kinect is adequate to build a inexpensive and comfortable system that classifies PD into three different stages related to FoG. Compared to the results of previous works, the obtained accuracy (93.40%) can be considered high. The relevant features for the classifier are: a) movement and position of the left arm, b) trunk position for slightly displaced walking sequences, and c) left shin angle, for straight walking sequences. However, we have obtained a better accuracy (96.23%) for a classifier that only uses features extracted from slightly displaced walking steps and spin walking steps. Finally, the obtained set of relevant features may lead to new rehabilitation therapies for PD patients with gait problems.

## Background

Parkinson’s Disease (PD) is a chronic neurodegenerative disease in which there is a dopaminergic deficit, due to the loss of nigroestriatal cells, causing motor impairment with tremor, rigidity and akinesia/bradykinesia as cardinal sign [1]. Gait impairment is among the motor problems associated with PD causing as significant disability that in advanced cases is not improved with the dopaminergic treatments [2].

Kinect ^*Ⓡ*^ is an innovative motion capture device developed by Microsoft [3] that is low-cost and constitutes a non-intrusive tracking device. With Kinect, it is possible to build systems without markers that allow users to control and interact with applications by using an interface that recognizes gestures, voice commands and objects without physical contact. In the literature, there exist some works that use Kinect whose goal is to provide solutions for people that have to perform some kind of rehabilitation, in general, and for people with Parkinson Disease (PD), in particular. Those systems are of three different types: a) systems that help in performing rehabilitation therapies, b) systems that help in the process of monitoring or evaluating users, and c) systems that try to help in the process of diagnosing PD.

There are several systems whose goal is to help in the rehabilitation processes, some of them not related to PD [4, 5] but others related to PD [6–9]. In [4] the authors present a prototype of a game-based telerehabilitation system that tries to prove the adequacy of using Kinect for telerehabilitation therapies. Kinerehab [5] is an occupational therapy system where patients can perform three different exercises: lift arms front, lift arms sides and lift arms up. In [6] they propose a game aimed at training dynamic postural control system for people with Parkinson Disease. In [7], they assess the effects on postural control of patients with Parkinson Disease when playing with Kinect. In [8] the authors present a Kinect-based virtual reality system that provides patients having Parkinson Disease (PD) with a motivating way to improve their rehabilitation. In [9] they present a Kinect-based system that can assess gait by identifying different gait features such as step length and states of the gait cycle by using a Finite-State Machine.

With respect to systems whose goal is to monitor or evaluate users, some works can be found in the rehabilitation area. In [10], D. González-Ortega et al. present a system for cognitive rehabilitation that tracks human body joints (head and hands) and the face and facial features (eyes, nose, and ears), achieving a successful monitoring percentage of 96.28%, using the depth information provided by the Kinect device. In [11] Antón et al. present a method that achieves a 91.9% accuracy in posture classification and 93.75% accuracy in trajectory recognition of rehabilitation exercises performed in front of Kinect. In [12] Clark et al. claim that Kinect can validly assess kinematic strategies of some postural control tests (forward reach, lateral reach and single-leg eyes-closed standing balance), and affirm that the results are comparable to those obtained with a 3D camera-based motion analysis system. The goal of the previous works is to accurately monitor postures of people that are standing in front of Kinect. However, the monitoring of the gait of people that suffer from Parkinson Disease (PD) is different because they are not standing in front of Kinect; they are walking towards Kinect or moving away from it. In that case, it is required that the Kinect sensor provides accurate depth data. In [13] Galna et al. compared the accuracy of the Kinect sensor with a 3D motion analysis system (Vicon) with 9 people with PD and 10 controls and concluded that Kinect could accurately measure some temporal and spatial features of clinically relevant movements but not for smaller movements such as hand clasping or toe tapping. In another similar work [14] where Kinect sensor accuracy was also compared with accuracy obtained with Vicon, but only with healthy people, Kharazi et al. concluded that Kinect could accurately track the knee and hip during the gait cycle, but not the ankle. In [15], Gabel et al. claim that Kinect sensor offers accurate and robust measurements of gait features including gait kinematics. However, more recently, in [16], Springer and Yogev present a review report of 12 similar studies that concluded that Kinect offers good validity for only some spatiotemporal gait parameters but poor validity for gait kinematics variables.

There are some systems that try to diagnose PD by using Kinect. In [17] Rocha et al. present a system to distinguish between PD patients and non PD people (4 PD patients and 5 non PD patients or controls which took part in the experiment) by using some measures extracted from the Kinect v2 such as velocity and acceleration of joints, distances and angles between joints, and other calculated measurements such as duration, length, average velocity and cadence of gait cycles. They identified the angle at the elbow as a significant feature to distinguish between PD and non-PD persons. In [18] Tupa et al. present a system to distinguish between PD patients, young healthy people and aged-matched people (18 PD patients, 18 aged-matched people and 15 students). Their classification algorithm obtained an accuracy of 97.2% by using some extracted gait features: leg length, normalized average stride length and gait velocity, all of them estimated with the data collected from Kinect. In [19] Eltoukhy et al. concluded that Kinect was sensitive enough to detect between group differences among PD and non-PD persons (11 non-PD and 8 PD) for some spatiotemporal and kinematics features such as stride length, stance and swing duration, gait and swing velocity. Moreover, they found these results were similar to those obtained with a 3D movement analysis system (BTS).

Moreover, there are also many systems whose goal is to diagnose PD by using other devices different from Kinect. Godinho et al. [20] present a review of 168 proposals that make use of 73 different devices (22 wearable that include sensors such as accelerometers, gyroscopes or magnetometers, 38 non-wearable that make use of force plates, infrared cameras, video recording cameras, ultrasounds, radio waves, etc., and 13 hybrid devices that make use of a combination of them). Among the gait parameters analyzed by those systems we can find: stride length and velocity, cadence gait cycle, swing phase, stance phase and double support ratio, trunk movements, number of steps, peak velocity, step time, symmetry of limbs, tremor, etc. These gait features can also be calculated with the data captured by Kinect, is a low-cost and less intrusive non-wearable device. It has to be said that 2 of the 9 recommended systems by Godinho et al. are also non-wearable devices. The first one [21], based on Nintendo Wii Balance Board, is used as an assessment tool for postural instability and not for gait, and it is considered a cheap and widespread device. The second one is the GAITRite system [22], an electronic pathway that contains pressure sensitive sensors and that provides information about gait features: walking speed, cadence and step length.

The goal of this work is to try to characterize the gait of PD patients by using the raw information collected from the Kinect device, and some features that can be calculated (or more correctly, estimated) from those raw data, knowing that this information is less precise than the information collected from more precise and sophisticated devices. With respect to this goal, it is known that in the early-moderate stage, the predominant form is short-stepped, with slow velocity and decreased amplitude of the segmental movement, which is related to hypokinesia [23]. Clinical characteristics of the locomotor pattern include reduced angular excursion of joints such as shoulder and knee [24] in one hemibody, which extend to the other hemibody as the disease progresses. Progressively, this gait becomes more abnormal and features such as flexion of the trunk, stopped posture, short steps, and shuffling, the latter associated with reduced ground clearance and festination, become more prominent [25]. Indeed, Freezing of Gait (FoG) is one of the most debilitating features of PD as it causes falls [26] and reduces mobility and quality of life [27]. It is described as “a brief, episodic absence or marked reduction of forward progression of the feet despite the intention to walk”. Furthermore, it is interesting to note that it seems to be associated with postural instability [28] but not with cardinal features of PD [29].

Moreover, we want to identify different stages of PD in order to detect the locomotion changes that progressively occur as the disease progresses. To achieve this, we will undertake a cross-sectional clinical study with three groups of patients: 1) early PD patients without axial impairment; 2) more evolved PD patients with higher gait impairment but without FoG; and 3) patients with advanced PD and FoG. To the best of our knowledge, this is the first work where such a clinical study has been done by using Kinect.

## Methods

In this section we present relevant information of the clinical study and the methods applied for the gait analysis of PD patients. Thus, we present the test scenario and the participants, the datasets about patients and Kinect recordings, the data preprocessing of the Kinect recordings and the feature selection and stage classification.

### Test scenario and participants

The test consisted of recording with Kinect the gait of the group of patients that took part in the clinical study, whose goal was to distinguish among the PD stage of patients. This test was performed in a corridor of the Hospital Donostia in San Sebastian during two different days (in November 2015 and January 2017).

The participants in the test were 30 patients^1^, of whom 8 were in the first stage of the disease (early PD patients without axial impairment), 11 in the second stage (more evolved PD patients with higher gait impairment but without FoG), and the remaining 11 in the third stage (patients with advanced PD and FoG). The stage of each patient was diagnosed by the neurologists of the Hospital Donostia.

The corridor was long enough for the patients to walk about 4.5-5.5 meters in both directions. They had to walk along the corridor forwards and backwards four times. Therefore, in total, each patient performed a walk of approximately 40 meters, including their corresponding 7 turnings or spins required to change the direction.

**Fig. 1:**
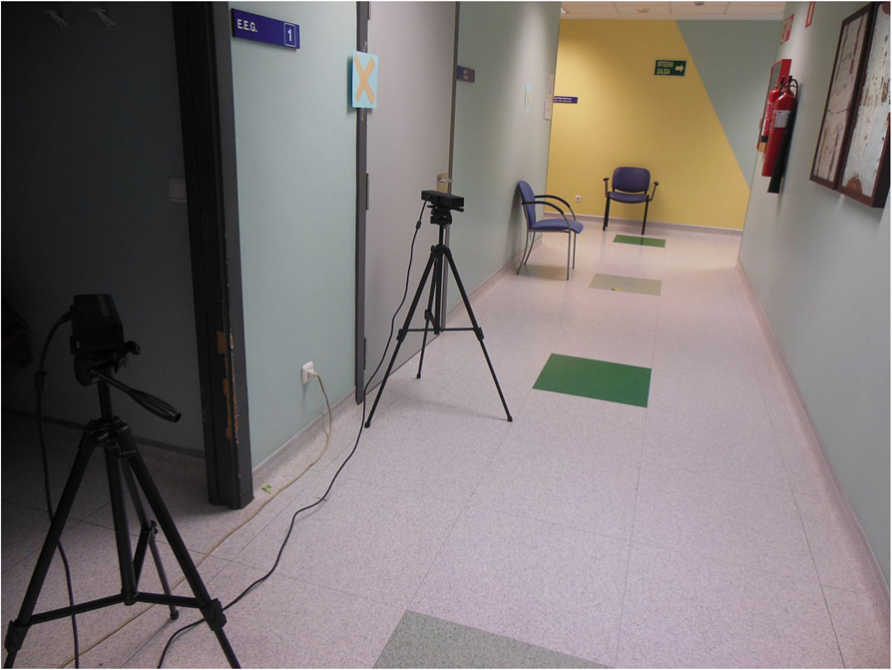
Real image of the test scene. In the image you can see the two Kinect cameras and the corridor through which the patients perform the walk

With the aim of covering the entire walk, including the spins that are very significant to identify PD patients, and also to capture the whole body of the patients (without cuts in the upper or below body), two Kinect devices were needed, each recording approximately 2.5 meters of the total route. The reason is that, with one Kinect device, only 2-3 meters can be covered. The two cameras were placed next to the wall^2^ (distributed as shown in Fig. 1), and tried to record simultaneously the complete walk. Each camera was connected to an independent computer that ran a data capture program that saved the data generated by the Kinect cameras during each patient walk. It is important to remark that there is a zone of overlap between the two cameras so that the walk can be recorded entirely, which also means that there is an area that is recorded twice (once for each camera).

### Dataset

The data available for the subsequent analysis is of two types: one is about clinical data of patients and the other one is the data captured with Kinect.

#### Dataset about patients

All patients were diagnosed according to UKPDS Brain Bank Criteria [31]. Each participant was also assessed on the Hoehn and Yahr staging and the Freezing of Gait Questionnaire [32]. None of the patients were deemed as having dementia according to Movement Disorder Society PD Dementia criteria [33]. All patients were assessed in the practically defined “off” state having stopped dopaminergic drugs 12 h before. This study was approved by the local ethics committee and written informed consent was obtained from all patients prior to study participation.

With respect to the clinical data of patients, we had the following information for each one: gender, age, weight, height, years of evolution of the PD, stage of the disease assigned by the neurologists (1, 2 or 3) based on the gait impairment as explained previously, and disease stage according to the Hoehn and Yahr (HY) scale [34], also assigned by the neurologists. As said before, 8 patients were assigned to the first stage of the disease, 11 to the second stage, and 11 to the third one, based also on the gait impairment assessment. According to the HY classification, there were 4 patients in stage 1.5, 7 in stage 2, 8 in stage 2.5, 10 in stage 3 and 1 in stage 4, with a strong Pearson correlation between the HY scale and the one used in the study: 0.793411. The first affected body side was also assessed for each patient. Table 1 shows the summary of patient information.

**Table 1:** Demographic and clinical characteristics

	PD stage 1	PD stage 2	PD stage 3	All stages
Number of patients	8	11	11	30
Age				
(mean/std)	68.73 / 6.49	70.56 / 5.69	72.57 / 6.72	70.8 / 6.27
Gender				
(#Male/#Female)	M: 5 F: 3	M: 11 F: 0	M: 9 F: 2	M: 25 F: 5
Disease years				
(mean/std)	3.08 / 2.36	7.04 / 6.59	11.15 / 4.63	6.68 / 5.86
HY scale				
(median)	1.75	2.5	3	2.5
(min/max)	1.5 / 2.5	2 / 3	2.5 / 4	1.5 / 4
FOG-Q total score				
(mean/std)	2.75 / 3.24	4.87 / 5.02	15.30 / 2.3	3.77 / 6.51
(min/max)	0 / 10	2 / 16	12 / 20	0 / 20
Initially affected hemobody				
(#Left/#Right/#Both)	L: 4 R: 3 B: 1	L: 7 R: 4 B: 0	L: 4 R: 5 B: 2	L: 15 R: 12 B: 3
Handedness				
(#Left/#Right)	L: 0 R: 8	L: 0 R: 11	L: 2 R: 9	L: 2 R: 28

#### Dataset of Kinect recordings

**Fig. 2:**
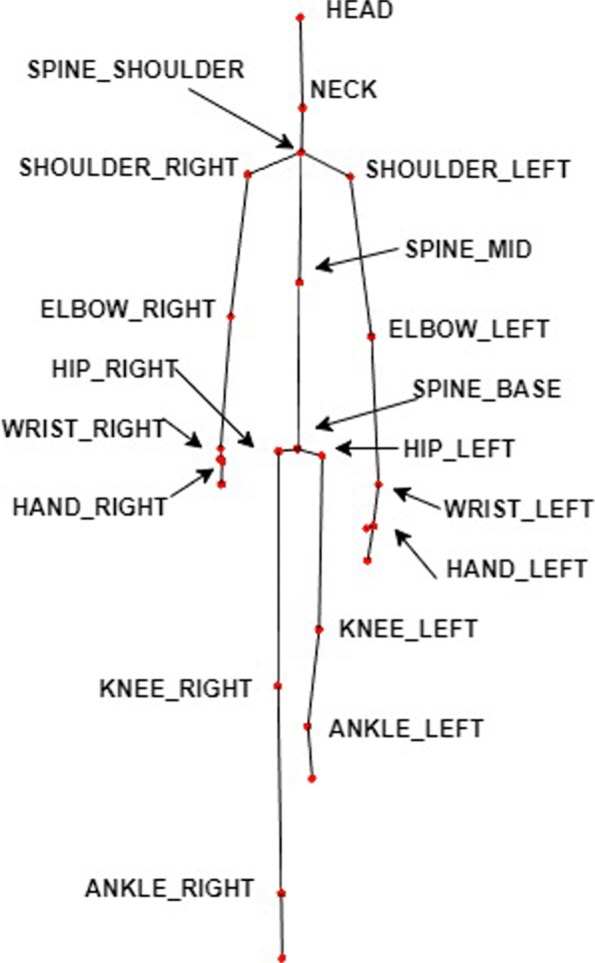
Kinect joints. Skeleton formed by the 19 most important joints of the 25 identified by Kinect

With respect to the data captured by Kinect from the patients, it can be said that each Kinect was capable of recording a maximum of 30 frames per second. The information that was saved for further processing was the following: depth images and skeletons. Regarding the size of the depth images, their dimension was 800 * 600 pixels. As to the skeleton structure, each frame is composed of 25 joints (distributed as shown in Fig. 2) from which the overall position of the skeleton of the frame is obtained and whether it is inferred by the Kinect or not. Nevertheless, only information of the joints (and not from the depth images) has been used for this work.

All joints are composed of 4 values, 3 of which indicate the joint position, with the fourth value indicating if the position value has been inferred by Kinect or not. A position value for a joint is inferred at a particular moment, if that joint is hidden for the infrared sensor of the Kinect camera, and the value is then calculated by the Kinect software by using previous or subsequent position values. If a joint position has been inferred, then the confidence of that data is low. The position values consist of the joint coordinates in X, Y and Z axes:

- The point X corresponds to the lateral (horizontal) position with reference to Kinect.
- The point Y corresponds to the vertical position with reference to Kinect.
- The point Z corresponds to the depth position, that is, the distance in depth from Kinect to the person.

**Fig. 3:**
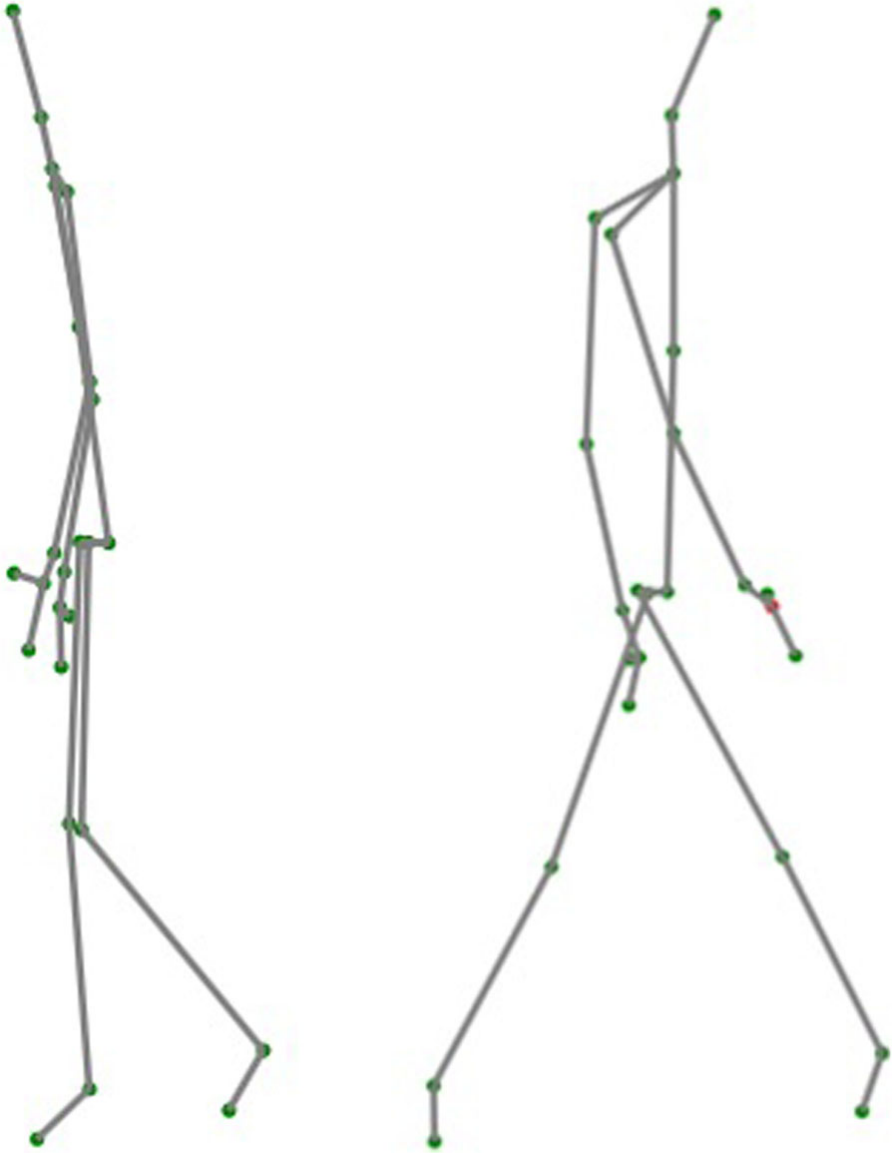
Structure of the skeleton according to the direction in which it walks. On the left, skeleton of a person who is walking towards the Kinect. On the right, skeleton of the same person who is walking in the opposite direction to the Kinect, that is, away from it

Apart from the mentioned Kinect feature of inferred joint positions, the Z value of the joints is not so accurate because Kinect has been mainly designed for people to stand in front of the camera (the depth position does not need to be so accurate) and not for people that walk in front of the camera. Related to this, it has to be said that when people are not looking at the camera or when they are moving away from the camera, Kinect tries to capture their skeletons as if they were in a position looking at the camera. The result is that the obtained data is of lower quality when patients walk away the camera; in Fig. 3, an impossible skeleton can be seen with the shoulders and the feet on the back side. It is interesting to know these facts about the quality of Kinect data so that an appropriate data cleaning can be performed during data processing.

### Data preprocessing: data cleaning and feature extraction

Before finding an accurate classifier, it is required to preprocess the raw data captured from Kinect with the goal of cleaning the data and extracting candidate features for the classifier. The preprocessing was performed according to the following steps: initial data cleaning, identification of gait steps, data cleaning of those steps, frames labeling, final data cleaning of steps, and feature extraction.

#### Initial data cleaning

In this first step, frames with many inferred points were removed because, as explained in a previous subsection, those values provide low reliability. The threshold to remove a frame was fixed on four inferred skeleton points.

#### Identification of gait steps

After the initial data cleaning, a gait step-based approach was followed to further reduce noise and to extract relevant features for the PD stage classification. A gait step is defined as a sequence of frames from the moment when one foot strikes the floor to the other foot striking the floor. A strategy based on local maximums of the distance between the feet time series was used in order to detect the gait steps in a sequence of frames. The frames registered between two consecutive local maximums were considered a gait step.

**Fig. 4:**
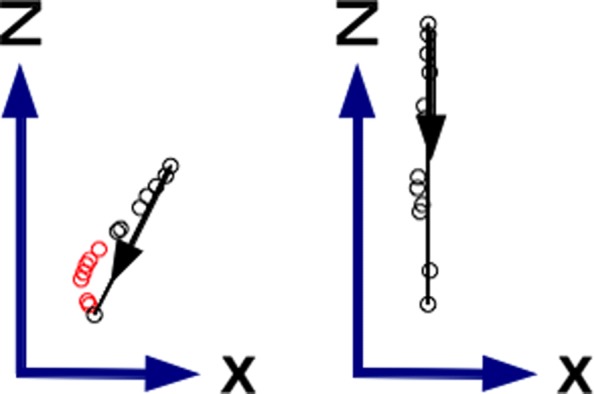
Spine base joint for two steps on XZ plan. Continuous line with arrow shows step displacement direction. Black dots belong to frames aligned with the displacement direction. Red dots belongs to frames not aligned with the displacement direction. Left step is a slightly displaced walking step. Right step is a straight walking step

The spine base joint on XZ plane (floor projection) of each two consecutive local maximums belonging to a valid step were used to calculate the displacement direction. Figure 4 shows the spine base joint on XZ plane time series for two steps. The right step in the figure represents a *straight walking step* (a step whose displacement direction angle is less than 0.1 radians from the straight line), with all its frames fully aligned with the displacement direction. The left step the Fig. 4 represents a *slightly displaced step* from the straight line (a step whose displacement direction angle is more than 0.1 radians from the straight line and less than 0.2 radians) and it may correspond to the beginning of a spin. Black dots in the figure correspond to frames aligned with the displacement direction (spine base less than 0.1 radians variation from the computed displacement direction). Red dots in the figure correspond to frames not aligned with the displacement direction (spine base more than 0.1 radians variation from the computed displacement direction).

Since the walking direction of a person is not always straight to the location of the camera (an example is provided in Fig. 4, left), an angular rotation over the y-axis (vertical axis) was applied in order to align the direction of walking of each person with the position of the Kinect sensor.

#### Data cleaning of steps

After identifying gait steps, another data cleaning process was carried out: each detected step was considered valid if only one foot was detected as moved. The frames corresponding to invalid steps were not considered for the next analysis step (frames labeling). At this stage, frames with a large difference in length between limbs of the same type were also removed for further analysis.

#### Frames labeling

After the data cleaning process of steps, it was attempted to categorize every frame as belonging to one of these types:

- straight walking step, frame aligned with the displacement direction, displacement towards the Kinect sensor
- slightly displaced walking step, frame aligned with the displacement direction, displacement towards the Kinect sensor
- slightly displaced walking step, frame equally aligned or not aligned with the displacement direction, displacement towards the Kinect sensor direction

At least 5 frames of the same type (a, b, c) had to be detected in the same step, in order to label such frames and to consider them for further analysis. This threshold was used in order to avoid noisy or outliers frames and it was empirically established.

#### Final data cleaning of steps

At this point, frames whose displacement direction was not towards the Kinect sensor were removed, unless they were identified as part of a spin. As it has been said in a previous subsection, data provided by Kinect when people are walking away the camera are not very accurate. However, when performing a spin it is important to maintain the data that may provide information about when the user is going towards or away from the camera.

#### Feature extraction

**Fig. 5:**
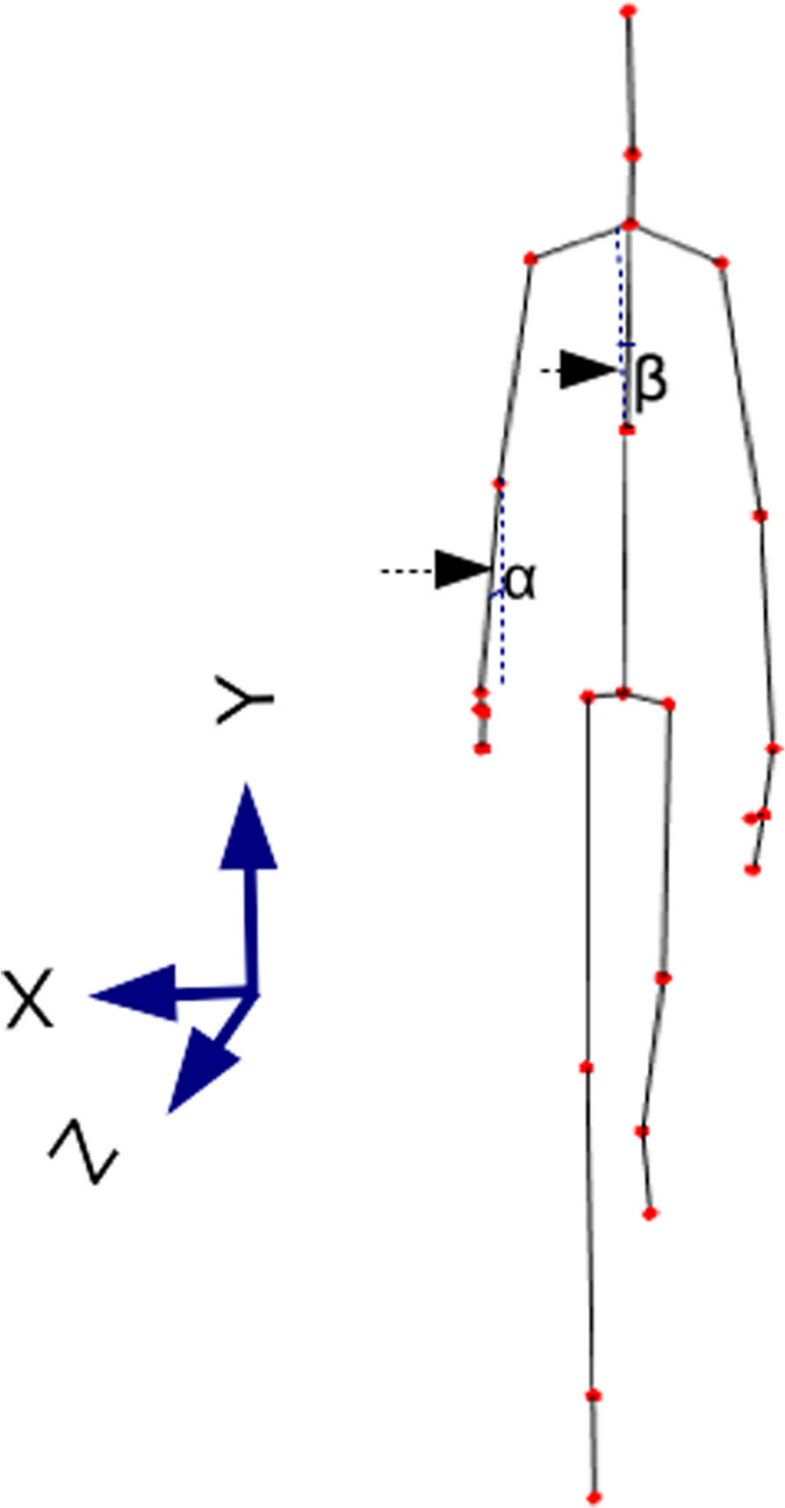
Kinect skeleton, XY view. Angle *α* corresponds to the right forearm angle projection on XY plane. Angle *β* corresponds to the lateral bent angle

**Fig. 6:**
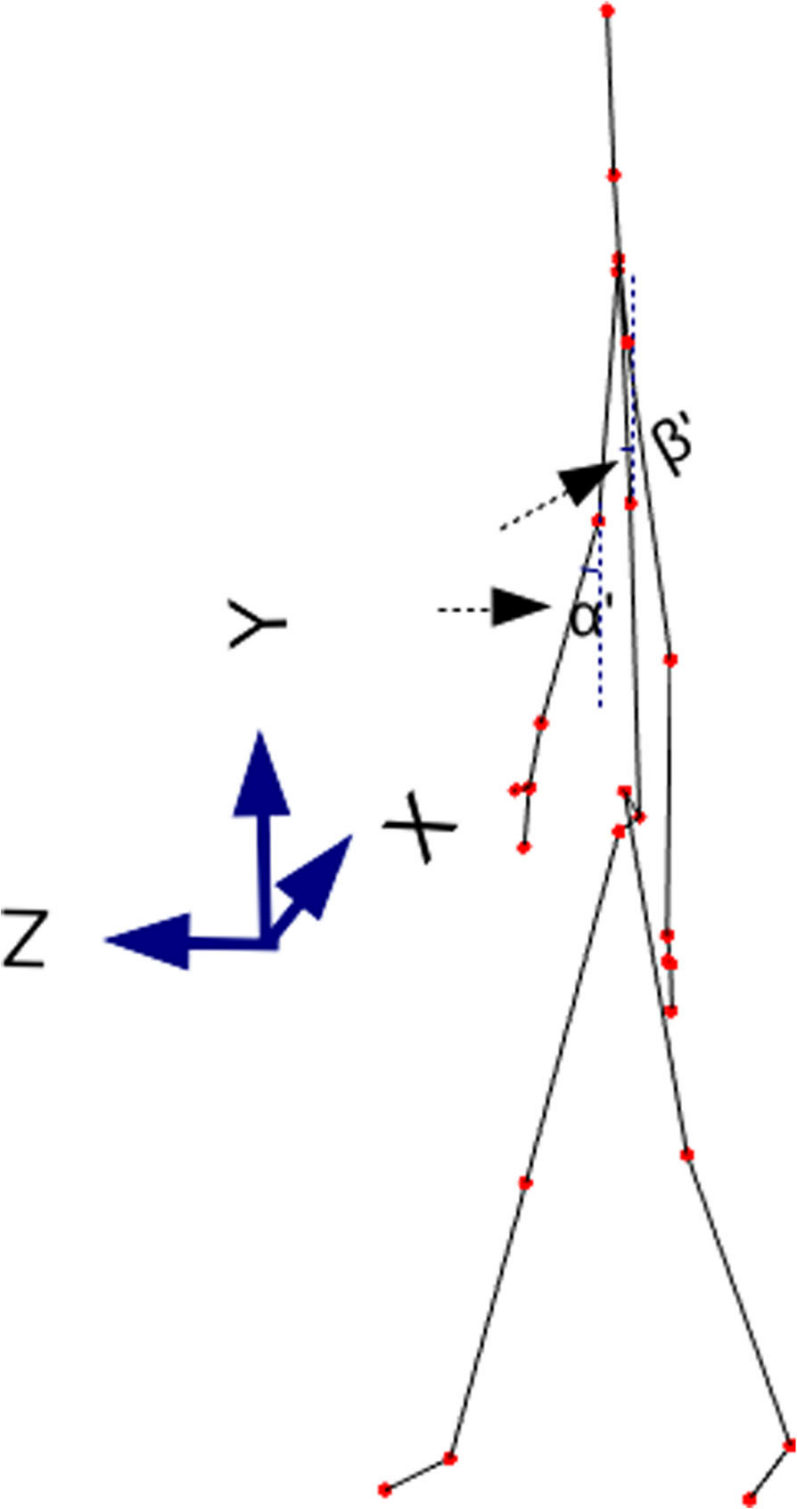
Kinect skeleton, YZ view. Angle *α*^′^ corresponds to the right forearm angle projection on YZ plane. Angle *β*^′^ corresponds to the frontal bent angle
- *Bent angles related to Parkinson disease*. Using the principles outlined in [36] we have calculated three body angles related to Parkinson disease. Namely, we calculated neck-flexion (NF), fore-bent (FB) and lateral-bent (LB) angles. Angle *β* in Fig. 5 and *β*^′^ in Fig. 6 show the lateral-bent and fore-bent, respectively. The LB and FB angles were computed using the spine-shoulder and spine-mid joints provided by Kinect. The NF angle was computed in a similar way to FB angle, using the neck and spine-shoulder joints provided by Kinect.

For each frame labeled as a, b or c, a set of angles was computed as follows:

- *Limbs angles*: angles of the projections on the XY and YZ plane (frontal and lateral plane, respectively) for each of the following bones: humerus, forearm, thigh, and shin, both left and right side. These measurements correspond to 16 angles. (4 limb types * 2 sides * 2 plane projection types = 16 angles). As an example of the computing of these angles, the angle *α* in Fig. 5 and *α*^′^ in Fig. 6 show the right forearm angle projection on xy and yz respectively. These kinds of measurements were successfully used previously for gait based recognition with a Kinect sensor [35] and we intended to test them here for the Parkinson stage classification.

A local regression (lowess) was applied to each angle series and then the mean and standard deviation were computed for each angle series. In this way, 19 mean angle-values were computed for each type of frames (a, b, c). These 57 values characterize postural states during the walking test. An additional 57 standard deviation values have been computed in a similar way. These values characterize the amount of variation of the corresponding angles during the walking test.

On the other hand, for *spin sequences*, the Kinect sensor was not intended to obtain sideways data. Still, we have extracted the *number of steps in spin* for those steps whose displacement direction was more than 0.2 radians from the straight line. As it has been said before, the displacement direction could be towards the Kinect sensor or moving away.

### Feature selection and stage classification

The features mentioned above (115 features, 38 for each a, b, c type of frame and 1 for spin steps) were computed for all the subjects (30 patients) recorded with Kinect. For classification purposes, the instances were balanced in the training set, which means that the instances were reweighted in the data so that each stage class had the same total weight.

There are different methods in the machine learning area that could be used to discriminate between PD stages. Several classification algorithms have been evaluated for each feature subset obtained previously. We evaluated different methods, for example, decision trees (the J48 algorithm provided by WEKA), Bayesian networks (BayesNet algorithm in WEKA), neural networks (multilayer perceptron algorithm in WEKA) and K-nearest neighbors classifier with (IBk algorithm in WEKA with *K*=1) among others. All the classification algorithms evaluated were run 100 times, using different random seed and a 10-fold cross validation schema. The performance of each classification method was measured in terms of accuracy (percentage of correctly classified instances). Additionally, the standard deviation over the different folds was computed. As the data was balanced, we did not consider another performance measure. All the evaluated methods were run by using the default WEKA parameters settings [37].

Once a classification method was selected, several experiments were carried out in order to improve the results of the classification method by selecting a better subset of features. Not all the computed features were relevant for the classification task and some of them could be redundant. In order to select the most relevant ones, we tested several filter methods: correlation-based feature selection (CFS), information gain and consistency subset evaluation. The filter methods have the advantage that they select variables regardless of the classification model and usually are robust to overfitting. All these methods were tested using the implementation provided by WEKA tool. Each filter method obtained a feature subset of a training set and the classification accuracy for that feature subset was computed over a test set. Once again 10-fold cross-validation was used an evaluation schema.

## Results

The list of experiments run in order to choose the best classification and feature selection method are summarized in Table 2. The first two columns in Table 2 identify the classification method. The third columns (*No feature selection*) shows the accuracy (average and standard deviation) of each classification method tested when no external feature selection algorithm is used. The last three columns show the accuracy of each classification method after applying the corresponding feature selection algorithm. Note that some classification methods (for example J48, Bayes Net) have their own built-in feature selection.

**Table 2:** Average accuracy and standard deviation (in parentheses) for the performed experiment

Weka classification method	Description	No feature selection	CFS	InfoGain	Consistency
J48	Decision trees	75.50	75.90	76.13	58.70
		(26.28)	(25.84)	(25.47)	(25.57)
PART	Rule based classifier	67.67	69.03	70.07	56.67
		(22.74)	(23.01)	(23.10)	(26.00)
Bayes Net	Bayesian netwoks	91.47	**93.40**	91.47	82.50
		(15.23)	(15.60)	(15.23)	(24.63)
Naive Bayes	Naïve Bayes classifier	54.23	79.40	75.43	62.20
		(25.61)	(23.16)	(23.53)	(27.58)
Multilayer	Neural netwoks	52.53	64.63	65.90	58.07
Perceptron		(26.93)	(25.69)	(24.70)	(27.12)
IBk	K-nearest neighbours	43.23	64.00	68.93	54.67
		(25.24)	(25.17)	(28.01)	(28.93)
Kstar	Instance-based learner	40.70	68.33	62.83	60.30
	using an entropic distance measure	(25.62)	(23.76)	(23.48)	(27.95)
SVM	Support vector machine	65.33	63.67	60.97	46.17
	(SVM) with C-SVM Type	(22.63)	(23.72)	(23.48)	(20.53)
SMO	SVM with sequential	64.37	67.80	68.23	49.93
	minimal optimization	(28.26)	(25.33)	(26.02)	(25.40)

Best accuracy appears in bold

One of the best feature selection methods turned out to be the Correlation-based Feature Selection (CFS), as can be seen in Table 2, fourth column. The CFS method considers both the individual predictive ability of each attribute and the degree of redundancy between them [38].

Table 3 shows the list of the features selected by the CFS algorithm, using data from all the subjects. Notice the small number of features (7) obtained by this method, compared to 115 features initially considered. It also highlights the fact that most of the selected features (5 features) are related to the spin execution, namely *F*_3_, *F*_4_ and *F*_5_ (related to type b frames), *F*_6_ (related to type c frames) and *F*_7_ (number of steps in spin).

**Table 3:** List of feature selected by CFS algorithm, using data from all the subjects

Feature name	Computing method	Angle name	Projection plane	Frames type
*F*_1_	Standard deviation	Left shin	YZ	a
*F*_2_	Standard deviation	Left humerus	XY	a
*F*_3_	Mean	Frontal bent	YZ	b
*F*_4_	Standard deviation	Lateral bent	XY	b
*F*_5_	Mean	Left forearm	YZ	b
*F*_6_	Standard deviation	Left humerus	XY	c
*F*_7_	Number of steps in spin	-	-	-

In terms of classification methods, Bayesian networks are the method that provided the better by far accuracy. Specifically, the accuracy obtained, using 10-fold cross-validation and CFS feature selection method, was ≈93.40*%* (see Table 2).

### Bayesian network model

**Fig. 7:**
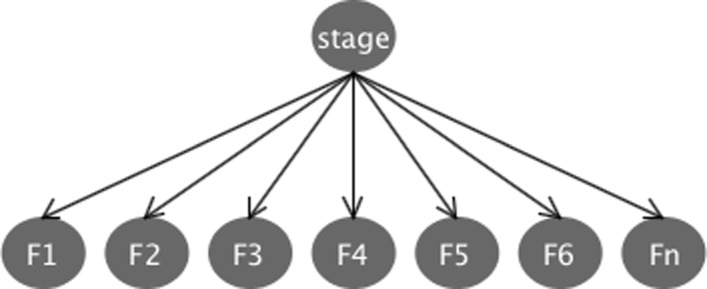
Bayesian network representation. Nodes correspond to PD stage and the selected features $F_{1}, F_{2}, \ddots F_{n}$ (*n*=7 for CFS feature selection method using data from all the subjects) and edges represent conditional dependencies

Bayesian network method [39] is a Directed Acyclic Graph (DAG), which is a graph with no cyclic paths. Each node in the DAG represents a random variable, while the edges between the nodes represent statistical dependencies between the corresponding random variables [40]. Figure 7 shows the structure of the Bayesian Network induced model for PD stage classification. The nodes correspond to the stage to be classified (1, 2 or 3) and the selected features (*F*_1_, *F*_2_, …, *F*_*n*_). Specifically, the CFS algorithm selects seven features related to PD stage to be classified. The arrows between stage node and *F*_*i*_ nodes indicate that a value taken by the variable stage depends on the values taken by variables *F*_*i*_ (with *i*∈[ 1..7] in the final Bayesian model obtained using data from all the subjects). These relationships are encoded by conditional probability distributions (CPDs) of the form *P*(*F*_*i*_|*stage*) (the probability of *F*_*i*_ given *stage*, as stage is parent for *F*_*i*_ nodes as may be observed in Fig. 7).

The CPDs of the final model (*P*(*F*_*i*_|*stage*)), obtained using the data of all the subjects, are shown and discussed below.

Table 4 shows the probability distribution for the *F*_1_ feature (standard deviation for left shin projection angle on YZ plane). The classification algorithm discretized the range of the *F*_1_ feature in two intervals: low for values in [0,0.462431) and high for values in [0.462431, +*∞*]. As may be observed, stages 2 and 3 are related to low values for this variable (that means *P*(*F*_1_=*low*|*stage*=3)=0.872) and *P*(*F*_1_=*low*|*stage*=2)=0.624). Stage 1 is strongly related to high values for this feature (*P*(*F*_1_=*high*|*stage*=1)=0.955). This feature characterizes the intensity of movement in depth of the left shin during straight walking test and we associate low values for this for this feature to small step length reported in Parkinson’s disease (Pearson correlation coefficient of this feature with step length is 0.60).

**Table 4:** CPD for *F*_1_ variable (standard deviation for left shin angle on YZ plane for type a frames)

*F*_1_/stage	Low	High
1	0.045	0.955
2	0.624	0.376
3	0.872	0.128

Table 5 shows the probability distribution for the *F*_2_ feature (standard deviation for left humerus angle on XY plane for frames of type a, straight walking). As may be observed, stage 3 is related to high values for this feature (*P*(*F*_2_=*high*|*stage*=3)=0.955), that means a higher intensity of the lateral movement of the left humerus. The high value was fixed in this case to the interval [0.048687, +*∞*]. This feature allows distinguishing the cases labeled as stage 3 from those labeled as stage 1 and 2.

**Table 5:** CPD for *F*_2_ variable (standard deviation for left humerus angle on XY plane for frames of type a)

*F*_2_/stage	Low	High
1	0.727	0.273
2	0.707	0.293
3	0.045	0.955

Table 6 shows the probability distribution for the *F*_3_ feature (mean frontal bent for type b frames). As may be observed, stage 1 is associated with low values for this feature (*P*(*F*_3_=*low*|*stage*=1)=0.587), that means low frontal bent, the subject walks upright, without leaning forward, stage 2 is associated with medium values and stage 3 to high values, respectively.

**Table 6:** CPD for *F*_3_ feature (mean frontal bent for type b frames)

*F*_3_/stage	Low	Medium	High
1	0.587	0.37	0.043
2	0.043	0.834	0.123
3	0.281	0.043	0.676

The *F*_4_ variable of the Bayesian network model is the standard deviation for lateral bent (LB) angle measured for frames of type b. In this case the CPF (see Table 7) associated stage 1 with low values. Stage 2 and 3 are associated with high values of *F*_4_. This feature allows distinguishing the cases in stage 1 from those in stage 2 and 3.

**Table 7:** CPD for *F*_4_ feature (standard deviation for lateral bent (LB) angle measured for frames of type b)

*F*_4_/stage *F*_4_	Low	High
1	0.955	0.045
2	0.211	0.789
3	0.128	0.872

The *F*_5_ feature is a postural one, namely the mean for the left forearm angle projected on YZ plane and measured for type b frames. In this case, the CPD related stage 3 to low values for *F*_5_ (0.789 probability, for more details, that means that the position of the left forearm is mainly vertical for type b frames, see Table 8). (slightly displaced walking steps, frame aligned with the displacement direction).

**Table 8:** CPD for *F*_5_ feature (mean for the left forearm angle projected on YZ plane and measured for type b frames)

*F*_5_/stage	Low	High
1	0.386	0.614
2	0.045	0.955
3	0.789	0.211

The *F*_6_ feature is the standard deviation for left humerus angle on XY plane for type c frames. This feature is similar to *F*_2_ feature. Stage 1 is mainly related to low values for this variable, stage 2 to medium values and stage 3 to high values, respectively (for more details, see Table 9).

**Table 9:** CPD for *F*_6_ feature (mean frontal bent for type b frames)

*F*_6_/stage	Low	Medium	High
1	0.587	0.043	0.37
2	0.043	0.913	0.043
3	0.043	0.518	0.439

The *F*_7_ feature is the number of steps for spin walking. In this case the CPF related the stage 3 mainly to high values (0.872 probability, that means that subjects of stage 3 are more likely to take many steps in spin, see Table 10). This feature allows distinguishing the cases in stage 3 from those in stage 1 and 2.

**Table 10:** CPD for *F*_7_ feature (number of steps for spin walking).

*F*_7_/stage	low	high
1	0.955	0.045
2	0.872	0.128
3	0.128	0.872

### Bayesian network classifier versus other classification methods

The Bayesian network induced in the previous subsection is very similar to a Naïve Bayesian classifier. In particular, a Naïve Bayes classifier may be seen as a specialized form of a Bayesian network with a structure similar to the one in Fig. 7, when depicted as a Bayesian network [41]. Therefore, differences in the classification results of Bayesian network and Naïve Bayes methods reported in Table 2 need further analysis.

When analyzing the two methods, we could conclude that the differences in the results are due to the fact that Bayesian network method discretizes the continuous values of the features used for classification. Discretization is the process of transforming a continuous-valued feature into a discrete one by creating a set of contiguous intervals that spans the range of the feature’s values, as has been done in the previous subsection with the selected features (*F*_1_, *F*_2_, …, *F*_7_). Indeed, when Naïve Bayes was used in conjunction with CFS method and feature discretization, the PD stage classification results obtained were similar to the ones obtained when using Bayesian network with CFS. Results over the impact of discretization over Naive-Bayesian classifier have been already observed in previous studies, such as [42] and the improvement of classification performance with discretization on biomedical datasets has been reported in literature in related works, such as [43].

In view of the improvements that the discretization can bring, several tests have been carried out in order to verify the performance for PD stages classification of other methods, when used in conjunction with feature discretization method. Among the classification methods initially tested in Table 2, Multilayer Perceptron, Ibk, Kstar and SVM improved their classification accuracy towards values similar to Bayesian network method when used in conjunction with feature discretization method.

As in this step we identified several methods with similar accuracy, we compared them using other criteria such as training and testing time. Table 11 shows the average training and testing time (calculated in milliseconds^3^) for each classification method with similar accuracy.

**Table 11:** Costs of the classification methods

Weka classification method	Training time (ms)	Testing time (ms)
Bayes net + CFS	9.26	0.03
Naïve Bayes +CFS + feature discretization	0.01	0
Multilayer perceptron + CFS + features discretization	55.01	0.04
IBk + CFS + feature discretization	9.19	0.05
Kstar + CFS + feature discretization	9.23	0.14
SVM + CFS + feature discretization	9.44	0.04

From the point of view of costs (see Table 11), Bayesian networks continue to be a very good option as they obtain low times for both training and testing. Nevertheless, Naïve Bayes with feature discretization overtakes Bayes Net method, especially in training times, since for Naïve Bayes structural learning is not required. Moreover, our obtained Bayesian network model is similar to a Naïve Bayes classifier with feature discretization because features are independent and not related among them. In any case, these two methods provide models which are easier to understand by a human than the other candidate methods, as have been seen in the previous subsection, through the network representation and the corresponding CPDs.

## Discussion

In this work, we have studied gait impairment in PD patients by using Kinect. As it has been said in “Background” section, systems that try to diagnose PD have used different gait features such as stride length and velocity, cadence gait cycle, swing phase, stance phase and double support ratio, trunk movements, number of steps, peak velocity, step time, symmetry of limbs or tremor. Moreover, by using Kinect, we have been able to experiment with other features derived from the data about joints captured with the Kinect device, and we have found that some of those features are relevant in the obtained Bayesian network model. In particular, the movement and the position of the left arm proved to be very relevant when classifying the PD stage. As may be observed in Table 3, the CFS algorithm selected three features related to the left arm position as relevant, that means almost half of the selected features. One of those features was a postural feature, related to positions held during slightly displaced walking steps (*F*_5_). The other two measures were the intensity of the lateral movement of the left humerus (*F*_2_ and *F*_6_). Moreover, the straight walking sequences provided useful information related to the intensity of movement of lower limbs (*F*_1_), meanwhile the slightly displaced walking sequences provided useful information related to trunk position (*F*_3_ and *F*_4_). We can also add that the spin and slightly displaced steps sequences of the test provided more useful information for classifying PD than the straight walking sequences. It is worth saying that although not all of the patients were initially affected in the same hemobody (left, right or both) (see Table 1), it seems that the left part of the body is somehow more affected, which may constitute an interesting and novel finding. However, as the patients were mainly right-handed, it cannot be concluded that similar results would be found with left-handed patients.

With respect to the biological/clinical significance of the selected features of the classifier, we can say that locomotion in FoG patients is characterised by (1) a profound and incremental decrease in stride length; (2) highly reduced joint ranges in the hip, knee, and ankle; (3) disordered temporal control of gait cycles and (4) high-frequency alternate trembling-like leg movements [27]. These gait deficits are unrelated to disease severity, and, although FoG has traditionally been viewed as a motor symptom of advanced PD, it does not correlate with the cardinal features of parkinsonism: rigidity, bradykinesia, and tremor [28]. However, it correlates with some characteristic cognitive domains decline such as executive dysfunction, set-shifting and conflict resolution. Although functional imaging studies have found primarily bilateral impairments in FoG in PD [44, 45], others have shown asymmetrical cortical functioning (i.e., hipoactivity in the right fronto-parietal cortices) [46] (Bartels *et al* 2008) and structural abnormalities in the right cortical hemisphere and brainstem [47] in FoG in PD, irrespectively of the side of basal ganglia (BG) degeneration. Because the right hemisphere plays an important role in monitoring sensorimotor information [48], it seems reasonable to expect that right cortical dysfunction in addition to right BG damage in FoG PD patients could exacerbate right hemisphere dysfunction. Thus, it might be expected that interruption of right hemisphere sensorimotor processes could lead to more severe gait impairments. Supporting this, recent research has found evidence that right BG damaged FoG PD patients also experience more FoG episodes in specific situations that demand increased sensory processing compared to left BG damaged FoG PD [49]. In these sense, our results show that the most relevant features in these patients in the classifier are morphological motor features related to previously reported right hemisphere impairment such as left shin, humerus and forearm angles. In addition, cholinergic neurons of the pedunculopontine nucleus (PPN) are particularly important for gait, as shown by posture and gait abnormalities induced in monkeys with cholinergic lesions in the PPN [50]. This is related to the involvement of frontal and lateral bents in our classifier, probably related to posture and balance impairment in these patients. Finally, in FoG PD patients it is known that a decrease in step length with shuffling becomes particularly apparent during gait initiation or turning [27], which will be represented by the number of steps during spin variable in the classifier.

Finally, it is difficult to compare our obtained accuracy (93.40%) with those obtained in other related works because, to the best of our knowledge, there are no similar Kinect-based systems that try to classify between different stages of PD. However, there are previous studies showing the reliability of Kinect distinguishing between healthy and PD patients [18, 20, 51, 52]. In our case, we have performed additional experiments with our system in order to classify between PD people and controls. We have tested our system with 11 healthy people, aged 65-70, and found that the accuracy of the classifier to distinguish between the 11 controls and the 30 PD people was 93.51%. This PD vs non-PD classifier has been created by using the Bayes Net method, after selecting features with CFS method. The training data have been balanced, and the experiments have been run 100 times. The set of relevant features for this classifier is different than for the classifier that distinguishes among the 3 PD stages, but that is normal because it is a different problem.

## Conclusion

In this paper, we have presented a Kinect-based system able of classifying between three different stages of Parkinson Disease related to severity of gait impairment. As Kinect is a low-cost and non-intrusive device, the system is less expensive and more comfortable than systems based on accelerometers, gyroscopes or exoskeletons. Moreover, the obtained accuracy of the classifier has shown that using Kinect is feasible to build such a system, although previous involving about Kinect presented some contradictory results.

The classifier was built with data captured by the system from a clinical study performed with 30 patients whose PD stage has been reported by neurologists. In the process of selecting the best classifier, different classification methods and techniques to select relevant features among the 115 features identified were tried. The best one, with an accuracy of 93.40% using 10-fold cross validation, has been a Bayesian Network classifier combined with a correlation-based feature selection method. The method obtains a model which is easily interpreted by humans. Feature discretization has been shown to improve the classification results of other methods that could also be suitable for the problem treated. In relation to the relevant features, it was found that almost half of them were related to the movement and the position of the left arm, most of the relevant features were related with spin performing and gait sequences with slightly displaced steps, and for straight walking sequences, some features were related to intensity of movement of lower limbs. Other classic features for the characterization of PD were also tested (the average length of the step, average speed, average cadence), but using them did not improve the classification results (although they have been found to have a certain degree of relevance for stage classification).

Moreover, this set of relevant features that correspond to specific body joints may lead to new rehabilitation therapies for PD patients with gait problems. Many studies have shown the efficacy of rehabilitation therapy at improving specific impairments and functional limitations in individuals with PD and FoG. Those rehabilitation therapies should try to improve the values for the obtained relevant features in PD patients with the goal of delaying as much as possible the progression from the initial stages of PD to the stage where FoG appears. Our Kinect-based system could be used in order to monitor the evolution of those PD patients.

We plan to perform prospective studies to analyse if the previously mentionated rehabilitation treatement delays the progression towards more severe forms of gait dysfunction.

## Abbreviations

CFS: Correlation-based feature selection
CPD: Conditional propability distributions
DAG: Directed Acyclic graph
FB: Fore-bent
FoG: Freezing of gait
HY: Hoehn And Yahr scale
LB: Lateral-bent
Max: Maximum
Min: Minimum
NF: Neck flexion
PD: Parkinson’s disease
SMO: SVM with sequential minimal optimization
STD: Standard deviation
SVM: Support vector machine
UKPDS brain bank: United Kingdom Parkinson’s disease society brain bank

## Footnotes

1 All the patients were carefully recruited by experienced neurologists according to UKPDS Brain Bank Criteria. In [30] they worked with 48 PD patients; in the other 17 works that we have checked they worked with less than 30.

2 In the test performed in November 2015, the angle of the Kinect camera was perpendicular to what should be a straight displacement of the patient. In the test of January 2017, the angle has been 60 degrees to what should be a straight displacement of the patient. The method presented in this paper is valid for different orientations of the Kinect cameras.

3 Each training and testing time is obtained as a mean over 100 runs. An iMac with 3,06 GHz Intel Core i3 processor has been used for the tests.
